# Rapamycin Response in Tumorigenic and Non-Tumorigenic Hepatic Cell Lines

**DOI:** 10.1371/journal.pone.0007373

**Published:** 2009-10-09

**Authors:** Rosa H. Jimenez, Joan M. Boylan, Ju-Seog Lee, Mirko Francesconi, Gastone Castellani, Jennifer A. Sanders, Philip A. Gruppuso

**Affiliations:** 1 Department of Pediatrics, Rhode Island Hospital and Brown University, Providence, Rhode Island, United States of America; 2 Department of Systems Biology, Division of Cancer Medicine, University of Texas M.D. Anderson Cancer Center, Houston, Texas, United States of America; 3 Interdepartmental Center “L. Galvani,” University of Bologna, Bologna, Italy; University of Washington, United States of America

## Abstract

**Background:**

The mTOR inhibitor rapamycin has anti-tumor activity across a variety of human cancers, including hepatocellular carcinoma. However, resistance to its growth inhibitory effects is common. We hypothesized that hepatic cell lines with varying rapamycin responsiveness would show common characteristics accounting for resistance to the drug.

**Methodology/Principal Findings:**

We profiled a total of 13 cell lines for rapamycin-induced growth inhibition. The non-tumorigenic rat liver epithelial cell line WB-F344 was highly sensitive while the tumorigenic WB311 cell line, originally derived from the WB-F344 line, was highly resistant. The other 11 cell lines showed a wide range of sensitivities. Rapamycin induced inhibition of cyclin E–dependent kinase activity in some cell lines, but the ability to do so did not correlate with sensitivity. Inhibition of cyclin E–dependent kinase activity was related to incorporation of p27^Kip1^ into cyclin E–containing complexes in some but not all cell lines. Similarly, sensitivity of global protein synthesis to rapamycin did not correlate with its anti-proliferative effect. However, rapamycin potently inhibited phosphorylation of two key substrates, ribosomal protein S6 and 4E-BP1, in all cases, indicating that the locus of rapamycin resistance was downstream from inhibition of mTOR Complex 1. Microarray analysis did not disclose a unifying mechanism for rapamycin resistance, although the glycolytic pathway was downregulated in all four cell lines studied.

**Conclusions/Significance:**

We conclude that the mechanisms of rapamycin resistance in hepatic cells involve alterations of signaling downstream from mTOR and that the mechanisms are highly heterogeneous, thus predicting that maintaining or promoting sensitivity will be highly challenging.

## Introduction

The Target of Rapamycin (TOR) is a nutrient-sensing kinase that is conserved from yeast [1] and Drosophila [2] to mammals. mTOR, the mammalian version of TOR, acts as a regulator of global translation, autophagy, ribosomal biogenesis, cell size, cell metabolism and gene expression [3]–[5]. Dysregulation of mTOR signaling contributes to the pathobiology of human cancer [6]–[8]. While activating mutations in mTOR itself have not been documented in cancer cells, modifications of upstream components that regulate mTOR and downstream effectors of the mTOR pathway have been observed.

Rapamycin was first identified as an antifungal agent [9] but was more recently shown to have immunosuppressive and chemotherapeutic properties [10]. Upon entering the cell, rapamycin binds its intracellular receptor FKBP12, which forms a complex with mTOR [5]. This interaction inhibits the kinase activity of mTOR, thereby blocking the phosphorylation of effector molecules, including p70 S6 kinase (p70S6K) and eukaryotic initiation factor 4E (eIF4E) binding protein 1 (4E-BP1) [5]. A consequence of mTOR inactivation in many cell types is inhibition of G1 progression [5].

Although rapamycin and its analogs show antitumor activity across a variety of human cancers, rapamycin resistance is a frequently observed characteristic of many cancers and cancer cell lines. Mechanisms of rapamycin resistance include mutations in FKBP12 and constituents of the mTOR pathway, including S6K1, 4E-BP1, p27^kip1^ and PP2A-related phosphatases [11]–[13]. However, these mechanisms do not necessarily account for all instances of rapamycin resistance.

In the case of hepatocellular carcinoma, initial clinical data came from patients who were placed on rapamycin or related drugs post-liver transplantation as immunosuppressive therapy [14]. The apparent salutary effect of these drugs was followed by the observation that activation of the mTOR pathway may be a predictor of poor prognosis [15], [16]. Several investigators have explored the mechanism by which rapamycin exerts anti-tumor effects on hepatocellular carcinoma [17]–[20], but studies on rapamycin resistance are lacking. Furthermore, data on the effects of mTOR inhibition on gene expression in cancer cells are extremely limited.

The starting point for our studies was a series of observations made using the *in vivo* models of liver regeneration and liver development [21]. While the former was highly sensitive to mTOR inhibition by administration of rapamycin to the whole animal, liver growth and hepatocyte proliferation in the late gestation fetal rat was not. We found that rapamycin administration to fetuses *in situ* potently inhibited mTOR signaling to ribosomal protein S6 phosphorylation, thus indicating that resistance could not be accounted for by factors directly involving mTOR activity [21]. Given the potential relationship between fetal development and oncogenesis, we proceeded to characterize a panel of hepatic cell lines, ranging from non-tumorigenic to highly tumorigenic, for their response to rapamycin. Our hypothesis was that these heterogeneous but related cell types would vary in their sensitivity to rapamycin with regard to cell proliferation, but that they would show common characteristics associated with resistance to the growth inhibitory effects of the drug.

## Methods

### Reagents

Rapamycin was purchased from LC Laboratories (Woburn, MA). The Quant-iT PicoGreen dsDNA Assay Kit were purchased from Invitrogen Corporation (Carlsbad, CA). Reagents for protein determination were obtained from the following sources: Bio-Rad protein assay kit, Bio-Rad (Hercules, CA); bicinchoninic acid (BCA) assay, Pierce Chemical Co. (Rockford, IL). Antibodies directed towards S6, phosphorylated S6 (Ser235/236), phosphorylated 4E-BP1 (Ser65 and Thr36/47), phosphorylated-Akt (Ser473 and Thr308) and total Akt were from Cell Signaling Technology, Inc. (Danvers, MA). Antibodies to 4E-BP1, cyclin E1, CDK2 and cyclin E1-agarose conjugate were obtained from Santa Cruz Biotechnology (Santa Cruz, CA). p27 antibody was from BD Biosciences (San Jose, CA).

### Cell Culture Conditions

Culture conditions have been described previously for the following cell lines: H4-II-E hepatoma cells [22]; GN5, GN6, GP6, GP7, GP7TB, and GN6TBC2 [23], [24]; WB-F344 [25]; WB311 [26]; the transplantable hepatocellular carcinoma (THC) 1682-C,1682-A and 252 cell lines [27]; THC H5D cell line [28]. Cells were plated at densities that would yield 60 to 80 percent confluence at the time of each experiment.

[^3^H]-thymidine incorporation into DNA was determined as previously described [29]. Previously described methods were employed for immunofluorescent detection of BrdU incorporation into DNA [30], flow cytometry [30] and determination of doubling times [31].

### Biochemical Analyses

For Western immunoblotting and cell cycle studies, cell lysates were prepared and analyzed as described previously [32]. Cyclin E1/CDK2 kinase activity was determined using immunoprecipitated proteins bound to antibody-Sepharose beads [32]. Protein synthesis was measured as the incorporation of [^3^H]-leucine into protein [33]. Cell lines were plated into six well plates (4×10^5^ per well) and allowed to attach overnight. Rapamycin (50 nM) and [^3^H]-leucine (1 µCi/ml) were added and allowed to incubate for 6 hr prior to cell lysis. Results were normalized to DNA content, which was measured using the fluorescent Quant-iT PicoGreen® dsDNA Assay Kit with λDNA (0–500 ng/ml) as standard.

### RNA Isolation and Microarray Hybridization

Cells were plated at 10^6^ cells per 100 mm plate and allowed to attach overnight. Vehicle (DMSO) or rapamycin (50 nM) was added for 24 hr. Total RNA was prepared from triplicate plates using TRIzol reagent (Invitrogen Corporation). For the WB-F344 and WB311 cells, gene expression was analyzed using the Affymetrix GeneChip® Rat Genome 230 2.0 Array (Affymetrix, Santa Clara, CA). Microarray fluorescence signals were normalized using Robust Multiarray Average [34]. For the GN5 and H5D cells, analyses were carried out with the RatRef-12 Expression BeadChips. The fluorescence signals were normalized using quantile normalization implemented in BeadStudio (Illumina Inc., San Diego, CA).

Affymetrix microarray data analysis was performed using the GeneSpring GX 7.3 software (Agilent Technologies, Inc., Santa Clara, CA). Illumina data analysis was performed using Partek® software, version 6.3 (Partek Inc., St. Louis, MO). For gene expression comparisons, genes with an average expression value less than 100 for both comparison groups for Affymetrix data or detection p-values >0.01 from BeadStudio for Illumina data, or fold-change less than 1.2 fold (up or down-regulated), were excluded from further statistical analyses. A two-sample t-test using p-value cut-off of 0.05 with multiple test correction (Benjamini and Hochberg false discovery rate) was applied for each gene to determine if the gene was differentially expressed in the pairwise comparisons [35]. Triplicate control and rapamycin samples for each cell line were used in these comparisons.

Hierarchical clustering for the Affymetrix and Illumina analyses was performed using GeneSpring 7.0 and GeneSpring R 2.0.1, respectively (Agilent Technologies). Clusters and heat maps were generated after median-centralization of log2-transformed gene expression data across all samples. Gene ontology and pathway terms (www.geneontology.org) were examined to identify biological themes associated with each set of genes regulated by rapamycin. Significantly altered genes from each comparison were classified into categories based on the gene ontology slim terms and mapped to the Kyoto Encyclopedia of Genes and Genomes (KEGG) Biopathway Database (http://www.genome.ad.jp/kegg/pathway.html). For the Fisher exact test, p-values were employed to determine the enriched gene ontology categories and overrepresented pathways. Data analysis was performed at the W. M. KECK Foundation Biotechnology Resources Laboratory (Yale University, New Haven, CT). The list of differentially expressed genes in each cell line was used for the network/pathway reconstruction analyses, which were performed as previously described [36].

The microarray studies described in this paper have been deposited in the NCI's Gene Expression Omnibus (GEO; [37]) and comply with the Minimal Information About a Microarray Experiment (MIAME) standard developed by the MGED Society (http://www.mged.org/Workgroups/MIAME/miame.html). The data may be accessed through the GEO SuperSeries accession number GSE17677 (http://www.ncbi.nlm.nih.gov/geo/query/acc.cgi?acc=GSE17677).

### Statistical Analyses

Data are shown as the mean and standard deviation. For data other than those derived from microarray analysis, differences between groups were assessed by one-way analysis of variance (ANOVA) with a Bonferroni post-hoc test using Prism 2.01 software (GraphPad Software, San Diego, CA). Chi square analysis was performed using standard methods.

## Results

### Cell Proliferation and Rapamycin Sensitivity

**Figure 1:**
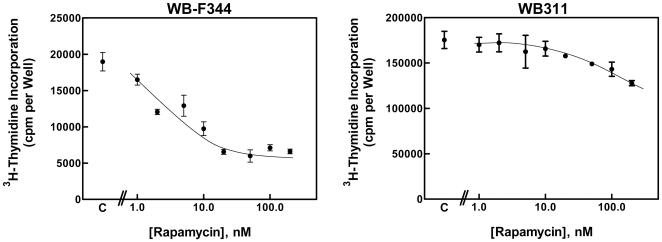
The effect of rapamycin on DNA synthesis in hepatic cell lines. WB-F344 cells (*left panel*) and WB311 cells (*right panel*) were exposed to vehicle (C) or rapamycin (0 to 200 nM) for 24 hr. [^3^H]-thymidine incorporation is shown as the mean ±1 standard deviation for triplicate determinations.

We first characterized the panel of hepatic cell lines for the effect of rapamycin on cell proliferation using incorporation of [^3^H]-thymidine into DNA. We initially focused on the WB-F344 cell line, the liver cell line from which many of the other cell lines studied were derived. A comparison of the WB-F344 cells with the spontaneously derived WB311 cells (Fig. 1) showed a marked difference in rapamycin sensitivity. Half-maximal inhibitory concentration (IC_50_) was approximately 10 nM for the WB-F344 cells and above 200 nM for the WB311 cells. The thymidine incorporation results were validated by immunohistochemical staining for BrdU incorporation. Nuclear labeling indices showed that the proliferation of the WB-F344 cells was inhibited by 30% with rapamycin (20 to 200 nM). WB311 cells were not affected. In flow cytometry studies, rapamycin increased the percentage of WB-F344 cells in G1 phase (66 to 76%) while the percent of cells in both the S (23% to 16%) and G2/M (11 to 8%) phases decreased. In contrast, there was no effect on cell cycle distribution of the WB311 cells. In neither case did rapamycin induce a hypodiploid peak as would have been expected with induction of apoptosis.

The thymidine incorporation analyses were extended to eleven other cell lines (Table 1). Response to rapamycin varied widely. IC_50_ values ranged from below 20 nM to above 200 nM. Percent inhibition at the highest rapamycin concentration tested (200 nM) ranged from 25 to 70%. The dose at which maximal inhibition was observed ranged from below 20 nM to 200 nM. These three indicators of sensitivity were correlated in some cases and not in others. For example, the H4-II-E cells (referred to as H4) and WB-F344 cells showed high sensitivity in all three measures. WB311 cells were uniformly resistant. In contrast, the GN5 and GN6 cells showed maximal inhibition at a low rapamycin concentration and a degree of inhibition that was significant. The THC H5D cells (referred to as H5D) showed an IC_50_ above 200 nM, but percent inhibition at 200 nM and the maximal inhibitory concentration both suggested relative sensitivity.

**Table 1:** Hepatic cell lines, mode of transformation, tumorigenicity and sensitivity to rapamycin.

Cell line	Mode of Transformation	Reference	Tumorigenic	Rapamycin IC_50_ (nM)	% Inhibition at 200 nM	Maximal Inhibition (Dose, nM)	Doubling Time (hr)
H4-II-E	Rat Hepatoma H35	[22]	No	2–5	70	10	24
WB-F344	None (parental)	[23]	No	10	68	20	42
GN5*	NNN, GGT-	[23]	Yes	20	55	20	21.6
GN6*	NNN, GGT-	[23]	Yes	20	63	20	
THC 1682-A	Choline-deficient diet	[27]	Yes	20	68	50	
GP7*	Solid Tumor, GGT+	[23]	Yes	50	59	100	
GN6TBC2*	Solid Tumor, GGT-	[24]	Yes	50	50	50	
THC 1682-C	Choline-deficient diet	[27]	Yes	100	67	50	
GP7TB*	Solid tumor, GGT+	[24]	Yes	100	53	50	
WB311*	Spontaneous	[26]	Yes	>200	25	200	15.5
GP6*	NNN, GGT+	[23]	Yes	>200	50	50	
THC 252	2-acetylamino-fluorine	[27]	Yes	>200	25	200	26.4
THC H5D	Azo dye	[28]	Yes	>200	40	20	26.6

Mode of transformation and tumorigenicity are based on published data. IC_50_ for rapamycin was determined by [^3^H]-thymidine incorporation. Maximal inhibition (dose) refers to the lowest concentration of rapamycin at which maximal inhibition of [^3^H]-thymidine incorporation was observed. NNN, N-methyl-N'-nitro-N-nitrosoguanidine; GGT, gamma-glutamyl transpeptidase; THC, transplantable hepatocellular carcinoma. *, derived from the parental WB-F344 cell line.

Doubling time was determined for six of the cell lines (Table 1). There was no correlation between doubling time and rapamycin sensitivity.

### Signaling Downstream and Upstream of mTOR

**Figure 2:**
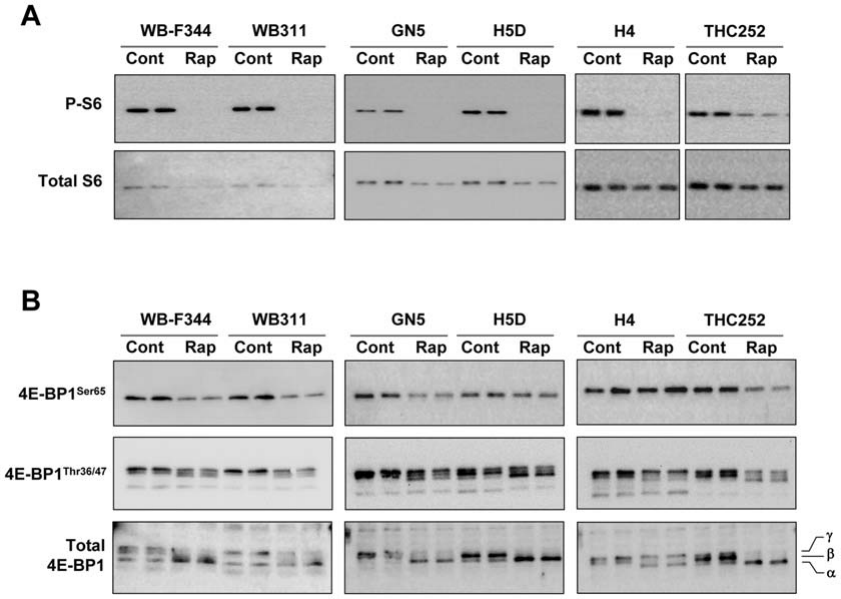
The effect of rapamycin on mTOR signaling in hepatic cell lines. *Panel A*: Duplicate cell lysates (5 µg protein) from cells with high sensitivity to rapamycin (WB-F344, H4), intermediate sensitivity (H5D, GN5) and resistance (WB311, THC252) were examined for phospho- and total S6 after exposure to DMSO or rapamycin (50 nM) for 24 hr. *Panel B*: A similar analysis was carried out for phosphorylated (Ser65; Thr36/47) and total 4EBP-1.

We examined the effects of rapamycin on the phosphorylation of mTOR targets in cell lines with high, intermediate and low sensitivity to rapamycin. The phosphorylation of S6 was abolished with rapamycin treatment in all cells (Fig. 2A). In all cases, rapamycin induced a modest decrease in the levels of total S6, but this was not sufficient to account for the loss of S6 phosphorylation. Rapamycin also induced a loss of 4E-BP1 phosphorylation at the mTOR-dependent Ser65 site in all cell lines except for H4 (Fig. 2B). Phosphorylation at Thr36/47 was sensitive in all cell lines (Fig. 2B). Analysis for total 4E-BP1 (Fig. 2B) showed a rapamycin-induced shift from the hyperphosphorylated β and γ forms to the hypophosphorylated α form in all cell lines. The S6 and 4E-BP1 findings were interpreted as indicating that mTOR signaling was sensitive to rapamycin in both sensitive and resistant cells and that the sensitivity of cells could not be accounted for by the ability of rapamycin to induce dephosphorylation of these mTOR targets.

**Figure 3:**
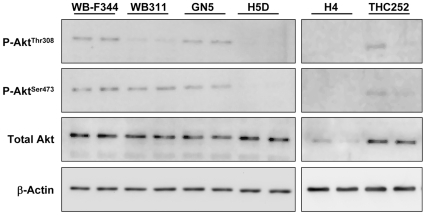
Akt phosphorylation and content in hepatic cell lines. Cell lysates were examined for phosphorylated (Ser308 and Thr437) and total Akt. β-actin was used as a control for loading and transfer.

We examined the basal Akt phosphorylation (Thr308 and Ser473) and content in several cell lines. Results (Fig. 3) revealed no correlation between rapamycin sensitivity and phospho-Akt. Total Akt abundance was similar among most cell lines with the exception of the H4 cells, which showed very low levels.

### Cell Cycle Control and Rapamycin Sensitivity

**Figure 4:**
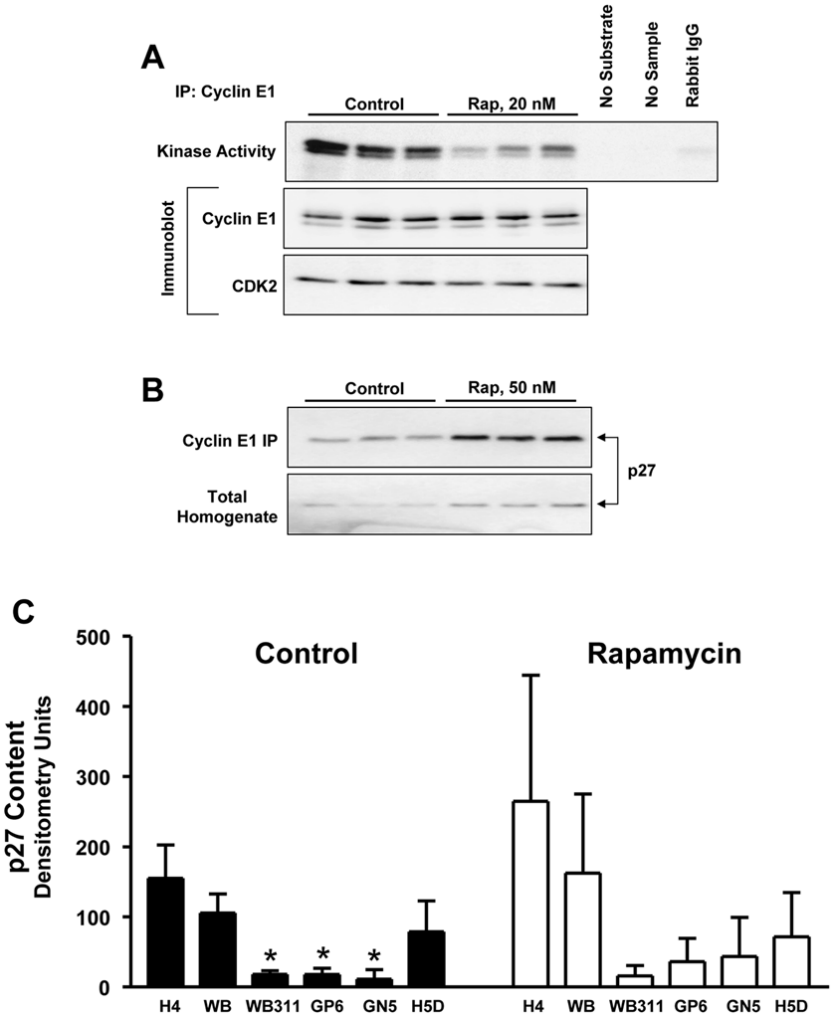
The effect of rapamycin on cyclin E1-containing complexes, cyclin E1-dependent kinase activity and levels of p27 in hepatic cells. *Panel A*: Triplicate cell extracts were prepared from H4 cells exposed to DMSO or 20 nM rapamycin for 24 hr. Extracts (200 µg) were immunoprecipitated with antibody to cyclin E1 and assayed for kinase activity using histone H1 as substrate. Controls for the activity assay included no substrate, no sample or immunoprecipitation with an irrelevant rabbit IgG. A separate immunoprecipitation was carried out to assess cyclin E1 and CDK2 in the immunoprecipitated complexes. *Panel B*: Triplicate cell extracts obtained as for *Panel A* were immunoprecipitated with cyclin E1 antibody followed by immunoblotting for p27. The same extracts were analyzed by direct immunoblotting for p27 content in the lysates. *Panel C*: Hepatic cell lines were examined for p27 content after exposure of the cells to DMSO (*filled bars*) or 100 nM rapamycin (*unfilled bars*) for 24 hr. Cell lysates (8.8 µg protein) were analyzed by direct immunoblotting for p27. p27 content is shown in the bar graph as density of the p27 signal for triplicate lysates (mean + standard deviation). *, P<0.05 versus H4 and WB by ANOVA. The p27 content was not affected by exposure to rapamycin for any cell line as determined by ANOVA.

We hypothesized that rapamycin responsiveness would be accounted for by rapamycin-induced changes in the expression or activity of cell cycle components involved in G1 progression. Initial studies were carried out on the highly sensitive H4 cells (IC_50_ below 5 nM). The kinase activity of cyclin E1/CDK2 complexes was determined after cyclin E1 immunoprecipitation using lysates made from H4 cells exposed to DMSO vehicle or 20 nM rapamycin for 24 hr. Kinase activity was decreased approximately 5-fold following rapamycin treatment (Fig. 4A). Rapamycin did not change the amount of either cyclin E1 or CDK2 protein in the complex (Fig. 4A).

We assessed the role of p27^Kip1^. Levels of p27 in H4 cell lysates showed a slight increase in response to rapamycin (Fig. 4B). The amount of p27 that was co-immunoprecipitated with cyclin E1 increased approximately 3.5-fold higher in response to rapamycin (Fig. 4B). Based on these results, we hypothesized that rapamycin resistance might be accounted for by low expression of p27. Lysates from cell lines cultured under basal conditions or exposed to 100 nM rapamycin for 24 hr were analyzed. Results showed that levels of p27 did not predict rapamycin sensitivity (Fig. 4C).

**Figure 5:**
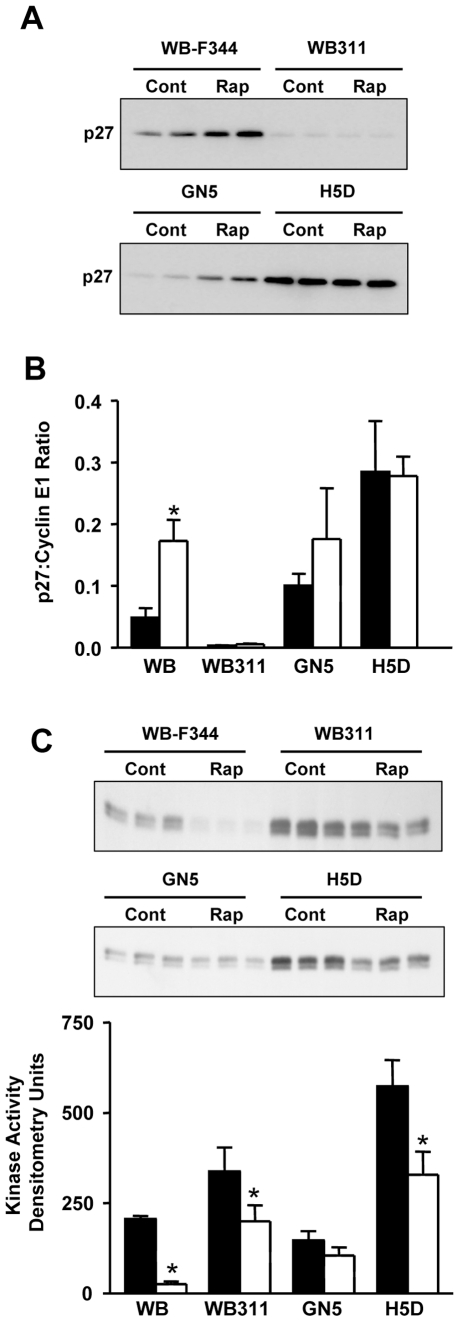
The effect of rapamycin on cyclin E1-associated p27 and cyclin E1-dependent kinase activity in hepatic cell lines. *Panel A*: Duplicate cell lysates (200 µg protein) were prepared from each cell line following exposure to DMSO or 100 nM rapamycin for 24 hr. Lysates were immunoprecipitated with cyclin E1 antibody and analyzed by immunoblotting for p27 content. *Panel B*: Triplicate lysates (100 µg protein) were immunoprecipitated with cyclin E1 antibody followed by immunoblotting for p27 and cyclin E1 levels. p27 content corrected for the amount of cyclin E1 in the immunoprecipitates is shown as the mean + standard deviation for DMSO (*filled bars*) or rapamycin (*unfilled bars*). *, P<0.005, control versus rapamycin by unpaired t-test. *Panel C*: Triplicate cell lysates from cells treated as above were immunoprecipitated with cyclin E1 antibody and assayed for kinase activity using histone H1 as substrate. The graph shows quantitation of the autoradiogram as mean + standard deviation for cells exposed to DMSO (*filled bars*) or rapamycin (*unfilled bars*). *, P<0.05 versus corresponding control as determined by ANOVA.

We examined the regulation of the cyclin E1-dependent kinase activity in the rapamycin sensitive WB-F344 cells, the resistant WB311 cells and two cell lines with intermediate sensitivity, GN5 and H5D. The ability of rapamycin to induce incorporation of p27 into cyclin E1 containing complexes was examined (Fig 5A). The WB-F344 cells showed a marked increase in the amount of p27 in the cyclin E1 complex upon exposure to rapamycin. A similar effect was seen in the faster growing GN5 cells. In contrast, WB311 cells showed very low levels of p27 while H5D cells contained high levels of p27 complexed to cyclin E1 in the basal state. Repeat analysis with triplicate samples, this time immunoblotting the immunoprecipitates for cyclin E1 and p27 (Fig. 5B), revealed that only the WB-F344 cells showed an increase in the amount of p27 relative to cyclin E1 in the immunoprecipitated complexes.

Triplicate immunoprecipitates prepared as for the above experiment were analyzed for cyclin E1-dependent kinase activity (Fig. 5C). The kinase activity of the WB-F344, WB311, and H5D cells were all significantly reduced upon rapamycin exposure. The GN5 cells exhibited minimal inhibition that was not statistically significant. The WB311 result was unexpected given that their rate of DNA synthesis was not inhibited by 100 nM rapamycin.

### Effect of Rapamycin on Global Protein Synthesis

**Figure 6:**
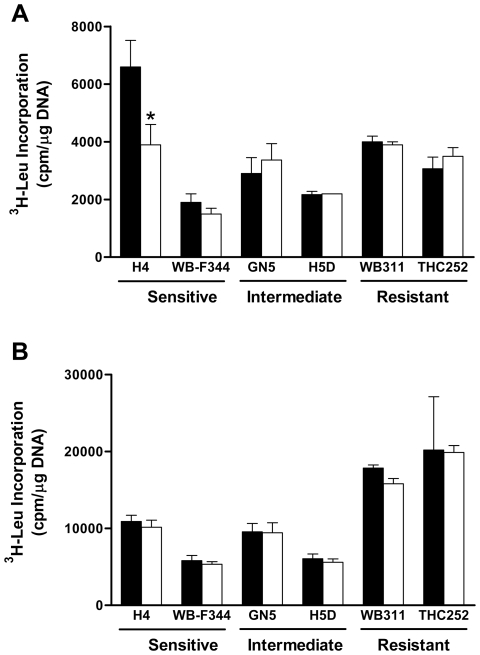
The effect of rapamycin on global protein synthesis in hepatic cell lines. Rapamycin sensitive cells (H4, WB-F344), resistant cells (WB311, THC252) and cells with intermediate sensitivity (GN5, H5D) were exposed to DMSO (*solid bars*) or rapamycin (*unfilled bars*) for 6 hr (*Panel A*) or 24 hr (*Panel B*). Protein synthesis was measured as incorporation of radiolabeled leucine into protein. Results, normalized per unit DNA, are shown as the mean + standard deviation for triplicate analyses. *, P<0.05 versus corresponding control as determined by ANOVA.

Given that rapamycin inhibits global protein synthesis in a spectrum of cell types [33], we hypothesized that inhibition of protein synthesis by rapamycin would correspond to its anti-proliferative potency. Cells were exposed to 50 nM rapamycin for 6 hr, during which they were also incubated with [^3^H]-leucine (Fig. 6A). Results were normalized to DNA content so as to reflect protein synthesis per unit cell number. H4 cells showed a decrease in protein synthesis of nearly 50% in response to rapamycin. However, none of the other cell lines studied showed an inhibitory effect. The experiment was repeated with a higher rapamycin dose and longer period of rapamycin exposure (200 nM for 24 hr). Results (Fig. 6B) showed an absence of rapamycin effect on incorporation of [^3^H]-leucine into protein in all cell lines, including H4 cells.

### Gene Expression Effects of Rapamycin in Hepatic Cells

**Figure 7:**
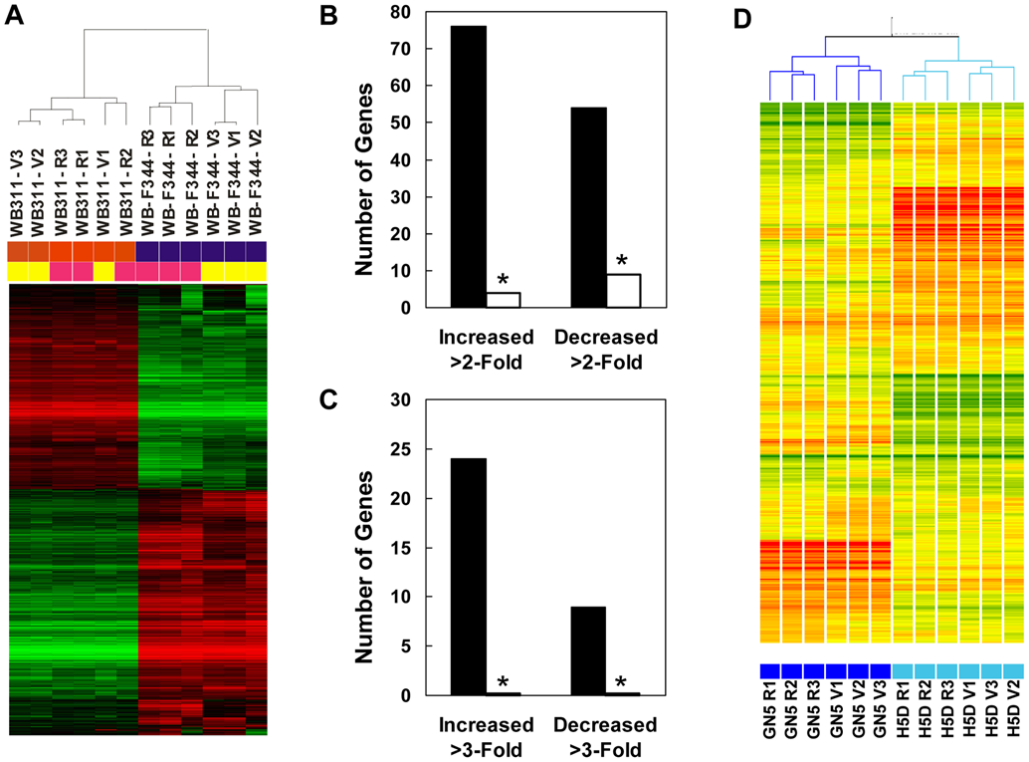
The effect of rapamycin on gene expression in hepatic cells. Cells were exposed to DMSO vehicle (V) or rapamycin (R; 50 nM) for 24 hr. RNA was prepared and analyzed by microarray using Affymetrix GeneChip® Rat Genome 230 2.0 Array for the WB-F344 and WB311 cells, or RatRef-12 Whole Genome Expression BeadChips for the GN5 and H5D cells. *Panel A*: Analysis of gene expression in the WB-F344 and WB311 cells. Red and green reflect high and low expression, respectively. A dendrogram of the cluster analysis is shown above the heat map. *Panel B*: The number of genes that were increased or decreased more than 2-fold is shown for the WB-F344 cells (*filled bars*) and the WB311 cells (*unfilled bars*). *, significant difference versus WB-F344 results as per chi square analysis. *Panel C*: Analysis for genes that showed a greater than 3-fold change. *Panel D*: Hierarchical clustering for the GN5 and H5D cells. Red and green reflect high and low expression levels, respectively. The yellow color represents genes that were unchanged. A dendrogram of the cluster analysis is shown above the heat map.

WB-F344 and WB311 cells were exposed to DMSO vehicle or rapamycin (50 nM) for 24 hr and processed for microarray analysis with Affymetrix GeneChip® Rat Genome 230 2.0 Arrays. Clustering analysis (Fig. 7A) showed that the WB-F344 and WB311 segregated based on the analysis of a total of 2,346 gene features that were significantly different across the four experimental groups. The dendrogram also showed that the control and rapamycin groups segregated for the rapamycin sensitive WB-F344 cells but not for the resistant WB311 cells.

In the WB-F344 cells, 2,034 probes (7.0% of total number of gene features on the Affymetrix chip) were differentially expressed in response to rapamycin. Of these, 679 were upregulated while 1,355 were downregulated. In the WB311 cells, 1,236 probes (4.4% of the total) were affected by rapamycin; 752 were upregulated and 484 downregulated. This difference in the magnitude of rapamycin effect was significant by chi-square analysis. The number of genes that showed a greater than 2-fold (Fig. 7B) or 3-fold (Fig. 7C) change confirmed that rapamycin effect on gene expression in WB-F344 cells was greater than in the WB311 cells.

We extended these analyses to two additional tumorigenic cell lines, GN5 and H5D, that displayed intermediate sensitivity to rapamycin. This allowed us to assess the effect of rapamycin independent of effects on cell proliferation. The GN5 cells and the H5D cells are fast growing relative to the WB-F344 cell line and slow growing compared to WB311 cells. As in the prior experiment, cells were exposed to DMSO vehicle or rapamycin (50 nM) for 24 hr. Analyses for this experiment, done at a different time than the analyses of the WB-F344 and WB311 cells, used Illumina RatRef-12 Whole Genome Expression BeadChips.

Clustering analysis of the results (Fig. 7D) showed that the GN5 and H5D cells segregated separately based on the analysis of a total of 1,293 gene features that were significantly different across the four experimental groups. Control and rapamycin groups segregated for both cell lines. In the GN5 cells, 777 probes (3.5% of the total number of genes on the chip) were affected by rapamycin (387 upregulated, 390 downregulated.) In the H5D cells, 636 probes (2.9% of the total) were affected (355 upregulated and 281 downregulated). The total number of genes altered in the GN5 cells and the number of genes showing a greater than 1.5-fold change were significantly greater than for the H5D cells. The list of all genes that showed differential expression in response to rapamycin for all four cell lines is provided in Table S1.

Gene ontology analysis (Fig. S1A–S1C) showed considerable variability across the four cell lines. However, several gene ontology terms related to cell metabolism, cell proliferation and cell death (Table S2) were affected in all of the cell lines studied. Pathway analysis (Tables S3A–S3C) revealed that genes involved in a number of biochemical and metabolic pathways were regulated in multiple cell lines. The glycolysis/gluconeogenesis pathway was uniformly affected with phosphoglycerol kinase-1 (Pgk1) being downregulated by rapamycin in all four cell lines. Pathway network reconstruction was performed to identify critical genes affected by rapamycin among the interphase of connected pathways (Fig. S2A–S2D). Results revealed that rapamycin did not target a specific pathway or gene across all cell lines. We did not identify underrepresented or overrepresented pathways that could account for rapamycin responsiveness.

## Discussion

The mTOR pathway is dysregulated in 40–50% of human hepatocellular carcinomas [14]. Owing to its potential as a chemotherapeutic agent, a substantial body of work has been devoted to understanding the mechanisms of resistance to the anti-proliferative effects of rapamycin and its analogs [11]–[13]. For our laboratory, the starting point for the present work was a series of in vivo studies showing that adult hepatocytes are sensitive to the growth inhibitory effects of rapamycin while late gestation fetal hepatocytes are resistant [21]. We hypothesized a relationship between fetal liver development and mechanisms involved in hepatic carcinogenesis. In order to test this hypothesis, we characterized the response to rapamycin among a panel of hepatic cell lines ranging from non-tumorigenic to highly tumorigenic. The goal was to identify a unifying mechanism that could be studied for its relevance to both fetal liver development and hepatic carcinogenesis. The ultimate goal was to extend our observations to liver cells that may represent precursors in the process of hepatic carcinogenesis.

A consistent finding across all cell lines was that the phosphorylation of S6 was sensitive to rapamycin. The effect on 4E-BP1 phosphorylation was less consistent but did not account for rapamycin sensitivity. This was particularly apparent in the marked effect on Ser65 phosphorylation in the WB-F344 and WB311 cells, which showed marked sensitivity and resistance with regard to DNA synthesis, respectively. In addition, the highly sensitive H4 cells showed no effect of rapamycin on phosphorylation at this site. This was unexpected given that only H4 cells responded to rapamycin with a significant inhibition of protein synthesis. The 4E-BP1 findings take on added significance with the recent development of potent mTOR kinase inhibitors that target the ATP binding site of mTOR. Studies using these inhibitors raised the possibility that rapamycin was exerting differential affects on S6 versus 4E-BP1 phosphorylation that could account for sensitivity to mTOR inhibition [13], [38]. This was not the case in our studies.

We have performed preliminary studies with one of these agents, Torin1 (data not shown). In studies on the WB-F344 and WB311 cell lines, we found that Torin1 and rapamycin had similar effects on 4E-BP1 and S6 phosphorylation but discrepant effects on DNA synthesis. The WB-F344 cells were sensitive to both agents, but the WB311 cells only showed significant inhibition of DNA synthesis in response to Torin1. These results support the conclusion from the present studies that the locus of rapamycin resistance in hepatic cells is downstream from mTOR complex 1 (mTORC1) but not from 4E-BP1 or S6 kinase. An alternative explanation is that pathways parallel to those involving mTORC1, possibly involving an mTOR complex other than mTORC1 or mTORC2, may account for resistance to rapamycin's antiproliferative effects. An example of such a mechanism may be the alternatively spliced form of mTOR termed mTORβ that was recently identified and assigned a role in the control of cell proliferation [39].

Our finding that Akt content did not correlate with rapamycin sensitivity is consistent with prior studies [40]. However, the same was true for phosphorylated Akt. Contrary to findings in other systems, Akt activation state, which may reflect “dependence” on mTOR signaling, was not a predictor of rapamycin response in the hepatic cells we examined [40].

Rapamycin induced the non-tumorigenic WB-F344 cells to arrest in G1, similar to other rapamycin sensitive cell types [5]. Our data in these cells indicated that incorporation of p27 into cyclin E1-containing complexes with resulting inhibition of cyclin E1-dependent kinase activity may have accounted for this G1 arrest. A corollary finding was the apparent deficiency of p27 in the highly rapamycin resistant WB311 cells. However, H5D cells, which displayed intermediate sensitivity, showed high levels of p27 in cyclin E1 complexes that were unaffected by rapamycin. Among the cell lines that showed a rapamycin-induced inhibition of cyclin E1-dependent kinase activity, there was evidence for both p27-dependent and p27-independent regulation of cyclin E1/CDK2 activity. These findings indicate heterogeneity across hepatic cells *in vitro* with regard to the effects of rapamycin on cyclin E1-dependent kinase activity.

It was striking that rapamycin induced short-term inhibition of leucine incorporation into protein in only one of the cell lines, the H4 cells. This was unexpected given the consistent effect of rapamycin on 4E-BP1 phosphorylation at the Ser65 and Thr36/47 sites, an effect that was absent only in the H4 cells. Furthermore, none of the cell lines showed inhibition of leucine incorporation into protein at 24 hr, a time point at which some cells showed growth arrest and 4E-BP1 phosphorylation was persistently inhibited. This is not the first circumstance in which 4E-BP1 phosphorylation in hepatic cells has been dissociated from activation of translation. This has been seen in liver regeneration [41], and our own laboratory made a similar observation in translation activation in the liver of rats refed following a period of starvation [42]. The finding that WB-F344 cells were highly sensitive to rapamycin but did not show sensitivity at the level of protein synthesis is particularly noteworthy. This result indicates that resistance to rapamycin-induced inhibition of protein synthesis cannot account for resistance at the level of DNA synthesis. Furthermore, the absence of an effect of rapamycin on the accumulation of protein incorporating radiolabeled leucine may indicate the absence of a rapamycin effect on autophagy. Such an effect would be expected to alter the steady state incorporation of radiolabeled leucine into protein. That being said, a direct examination of the effects of rapamycin on autophagy in these hepatic cell lines may be warranted.

The role of TOR in the regulation of gene transcription has been mostly characterized in yeast [43], [44] where the nuclear localization and the activity of several nutrient and stress-responsive transcription factors are regulated by mTORC1-dependent phosphorylation [45]. In mammalian cells, mTOR-mediated transcriptional control has been associated with regulation of the expression of relatively few genes. The genes that are best characterized for rapamycin responsiveness include Polymerase I, IGF-II and ribosomal DNA [46]–[48]. We undertook a broader analysis of rapamycin effect on gene expression so as to identify genes, groups of genes (ontology analysis) or pathways that could account for rapamycin responsiveness. We observed that genes associated with the mTOR pathway were generally not affected at the mRNA level by rapamycin in either sensitive or resistant hepatic cell lines. In addition, none of the known effectors and downstream targets of TOR in yeast, such as Gln3, Ure2 and Tip41 [44], were affected. Most importantly, the genes that were modulated in the sensitive WB-F344 cells but not the resistant WB311 cells included very few candidates that could account for rapamycin resistance at the level of cell cycle control, cell proliferation, translation or apoptosis. The differential expression of genes induced by rapamycin was greatest in the most sensitive WB-F344 cells. While this might be consistent with an impaired effect of rapamycin on gene expression in resistant cells, it may well be accounted for by the absence of rapamycin-induced growth inhibition in the resistant cells. It was striking that the genes most potently affected in the sensitive cells were, in general, not associated with cell growth. Finally, we identified several rapamycin-affected genes that have not previously been considered rapamycin sensitive. In particular, the gene encoding phosphoglycerol kinase-1, an enzyme in the glycolytic pathway, was downregulated in response to rapamycin in the four cell lines tested.

Despite the highly heterogeneous response to rapamycin seen in the panel of hepatic cell lines that we studied, a uniform finding was that rapamycin was effective in blocking mTOR signaling to S6 and 4E-BP1 in all cell lines. The precise effect of rapamycin on 4E-BP1 phosphorylation varied but could not account for rapamycin response. More importantly, cell proliferation, cell cycle regulation at the level of cyclin E-dependent signaling, global protein synthesis and gene expression were heterogeneous among the different cell lines. We deliberately chose related hepatic cells with diverse characteristics to identify rapamycin response without the bias inherent in studying any single cell line. Our observations lead to the conclusion that different tumorigenic hepatic cell lines possess mechanisms of rapamycin resistance that are not dependent on alterations in the direct actions of mTOR on its targets. Resistance to the anti-proliferative actions of this drug seems to depend on dysregulation of G1-to-S progression but not a specific mode of cell cycle dysregulation. However, mechanisms of rapamycin resistance at the level of the cell cycle vary, implying a level of complexity that will present challenges to the efficacy of rapamycin in the treatment of liver cancer.

## Supporting Information

Figure S1 Gene Ontology Analysis. Panel A. Gene ontology biological process terms responsive to rapamycin (50 nM, 24 hr) in hepatic cell lines. The bars indicate the number of differentially expressed genes per cell line for each significant Gene Ontology (GO) term sub-category. C, cell growth and/or maintenance; M, metabolism; CM, cell communication; D, development; NNNNAM, nucleobase, nucleoside, nucleotide and nucleic acid metabolism; PM, protein metabolism; T, transport. Panel B. Gene ontology molecular function terms responsive to rapamycin (50 nM, 24 hr) in hepatic cell lines. The bars indicate the number of differentially expressed genes per cell line for each significant Gene Ontology (GO) term sub-category. NAB, nucleic acid binding; CA, catalytic activity; TA, transporter activity; TFA, transcription factor activity; B, binding. Panel C. Gene ontology cell component terms responsive to rapamycin (50 nM, 24 hr) in hepatic cell lines. The bars indicate the number of differentially expressed genes per cell line for each significant Gene Ontology (GO) term sub-category. CI, cell:intracellular; C, cytoplasm; EES, external encapsulating structure.
(0.42 MB PPT)

Figure S2 Bipartite Network of Pathways Analysis. Panel A. Bipartite network of pathways and genes representing targets of mTOR in WB-F344 cells. Pathways are represented by circles and genes by squares. Gray circles indicate pathways that were not significant while red and blue circles indicate significant pathways that are overrepresented and underrepresented, respectively. The intensity of the squares with green shading indicates the degree of pathway membership ranging from light green (genes connected to few pathways) to dark green (the hubs). Pathway sub-networks are related to bladder cancer, TGF-beta signaling, tight junction, metabolism and aminoacyl-tRNA biosynthesis biological functions. The Akt, Prkca, Ccnd1 and myc genes were connected to significant pathways. Panel B. Bipartite network of pathways and genes representing targets of mTOR in WB311 cells. Symbols are those used for Figure 1A. Pathway sub-networks are related to metabolism and cellular biological functions. The Ccnd1 and myc genes were connected to pathways that were not significant. Panel C. Bipartite network of pathways and genes representing targets of mTOR in GN5 cells. Symbols are those used for Figure 1A. Overrepresented pathway sub-networks were not identified, while the neuroactive ligand receptor interaction pathway was underrepresented. The Nfkbia, Hsd3b7, Tgfb2, Tgfb3, Tgfa, and Maoa genes were connected to pathways that were not significant. Panel D. Bipartite network of pathways and genes representing targets of mTOR in H5D cells. Symbols are those used for Figure 1A. The neuroactive ligand receptor interaction pathway was underrepresented. Overrepresented pathway sub-networks are related to metabolism, Alzheimer's disease, melanogenesis, thyroid cancer and prostate cancer. The Mapk3 gene was connected to pathways that were not significant.
(0.87 MB PPT)

Table S1 Genes that show differential expression in response to rapamycin. Results are shown for the WB-F344 (Table S1A), WB311 (Table S1B), GN5 (Table S1C) and H5D (Table S1D) cell lines. Note that for Tables S1A and S1B, some genes have more than one Affymatrix Probe ID while some have neither a gene title nor a gene symbol.
(0.80 MB XLS)

Table S2 Gene ontology terms and associated genes sensitive to rapamycin among all four hepatic cell lines tested. MF, molecular function; BP, biological process. In the Probe ID column, the top number is the Affymetrix platform ID and the bottom is the Illumina platform ID.
(0.04 MB DOC)

Table S3 Pathways affected by rapamycin in hepatic cell lines. Table S3A. Results are given for the WB-F344 and WB311 cell lines (Table S3A) and for the GN5 and H5D cell lines (Table S3B). Grey highlighting denotes pathways common to all cell lines studied. Table S3C. Effect of rapamycin on genes in the glycolysis/gluconeogenesis pathway. Results are given for all four hepatic cell lines studied.
(0.10 MB DOC)
