# Supplementary figures and images for: Rapamycin Response in Tumorigenic and Non-Tumorigenic Hepatic Cell Lines
Source: PLoS One. 2009 Oct 9;4(10):e7373. doi: 10.1371/journal.pone.0007373 (PMC2756589; doi:10.1371/journal.pone.0007373)

## Slide 1
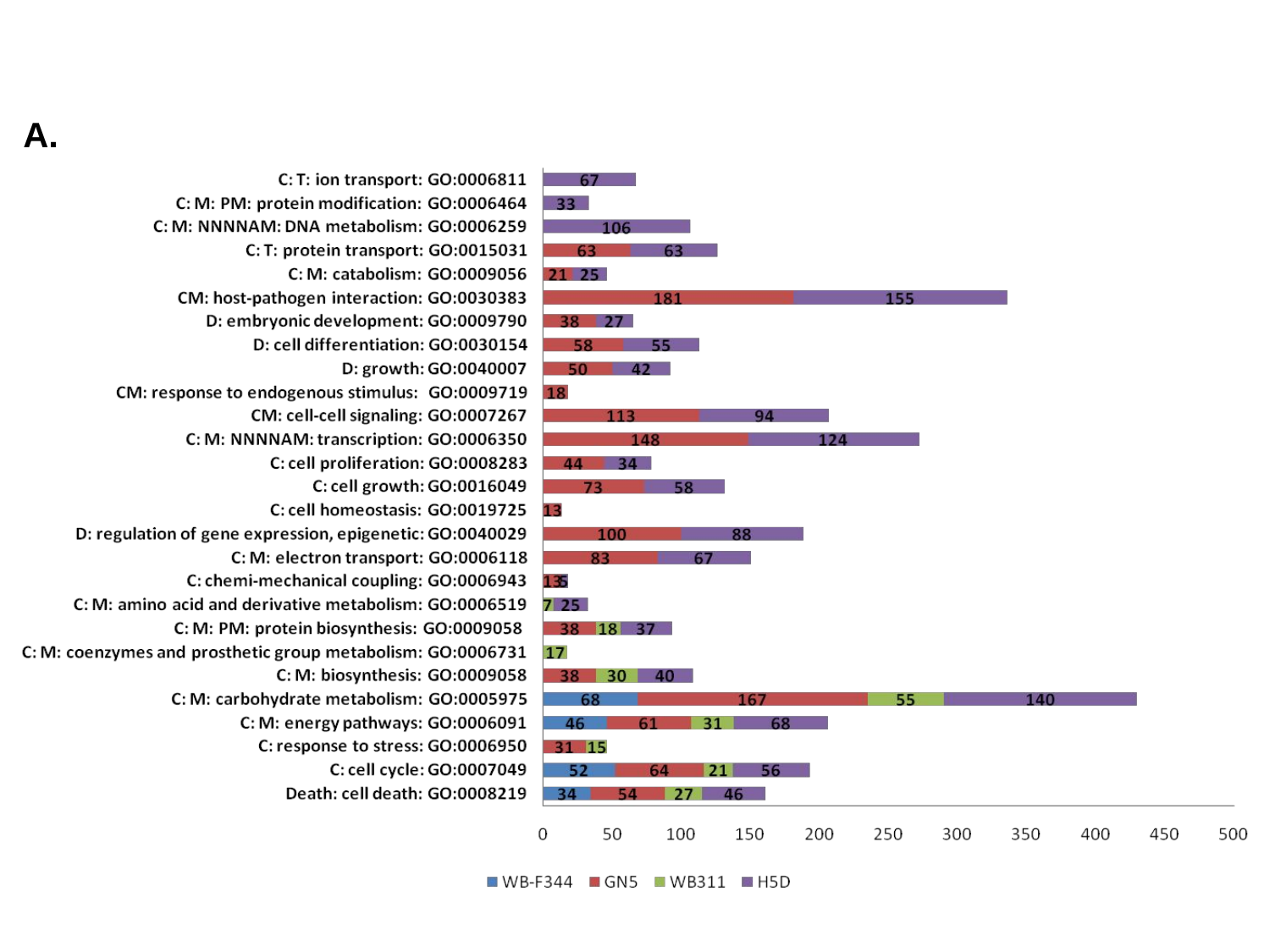

# A.

## Slide 2
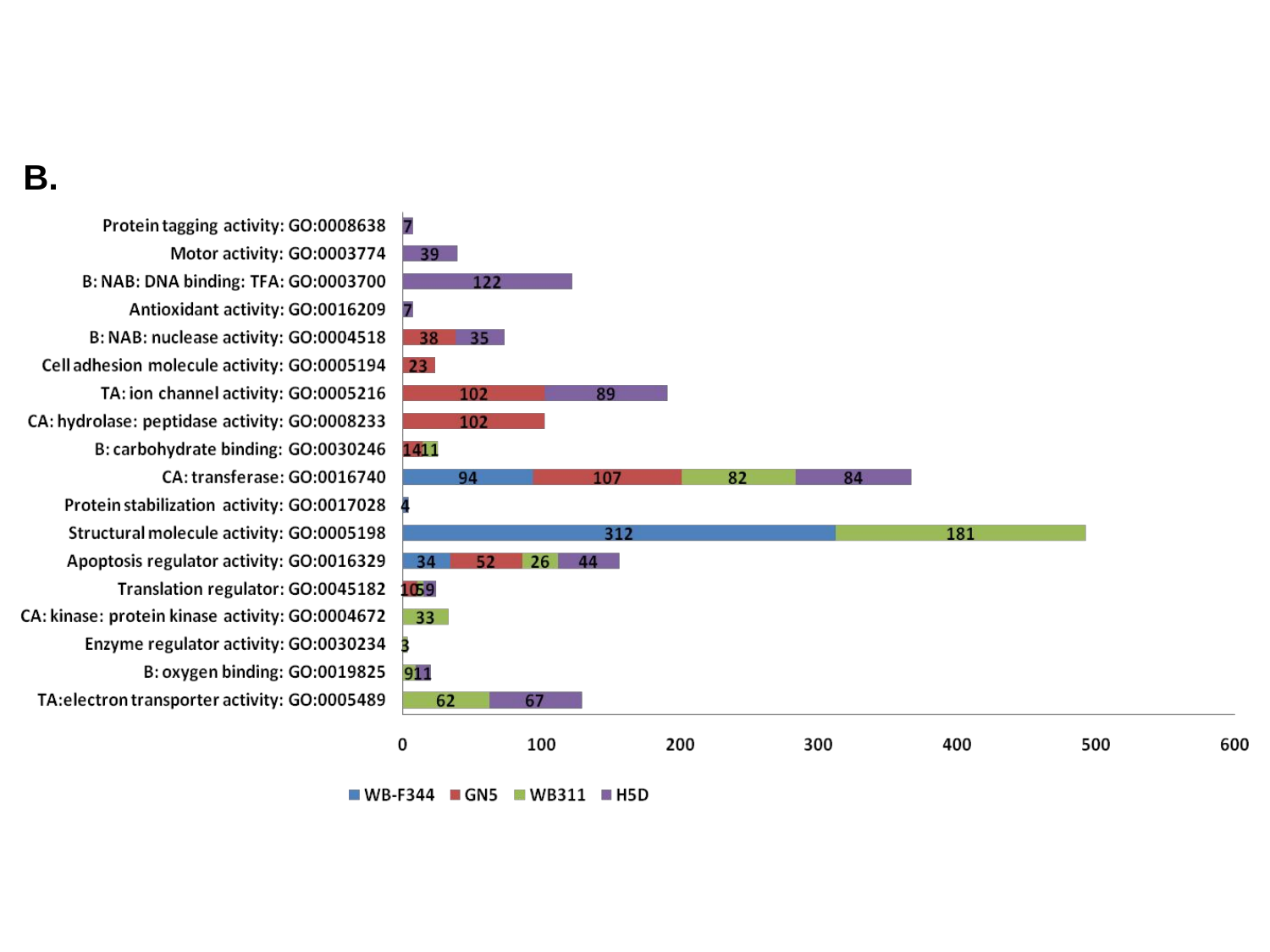

B.

## Slide 3
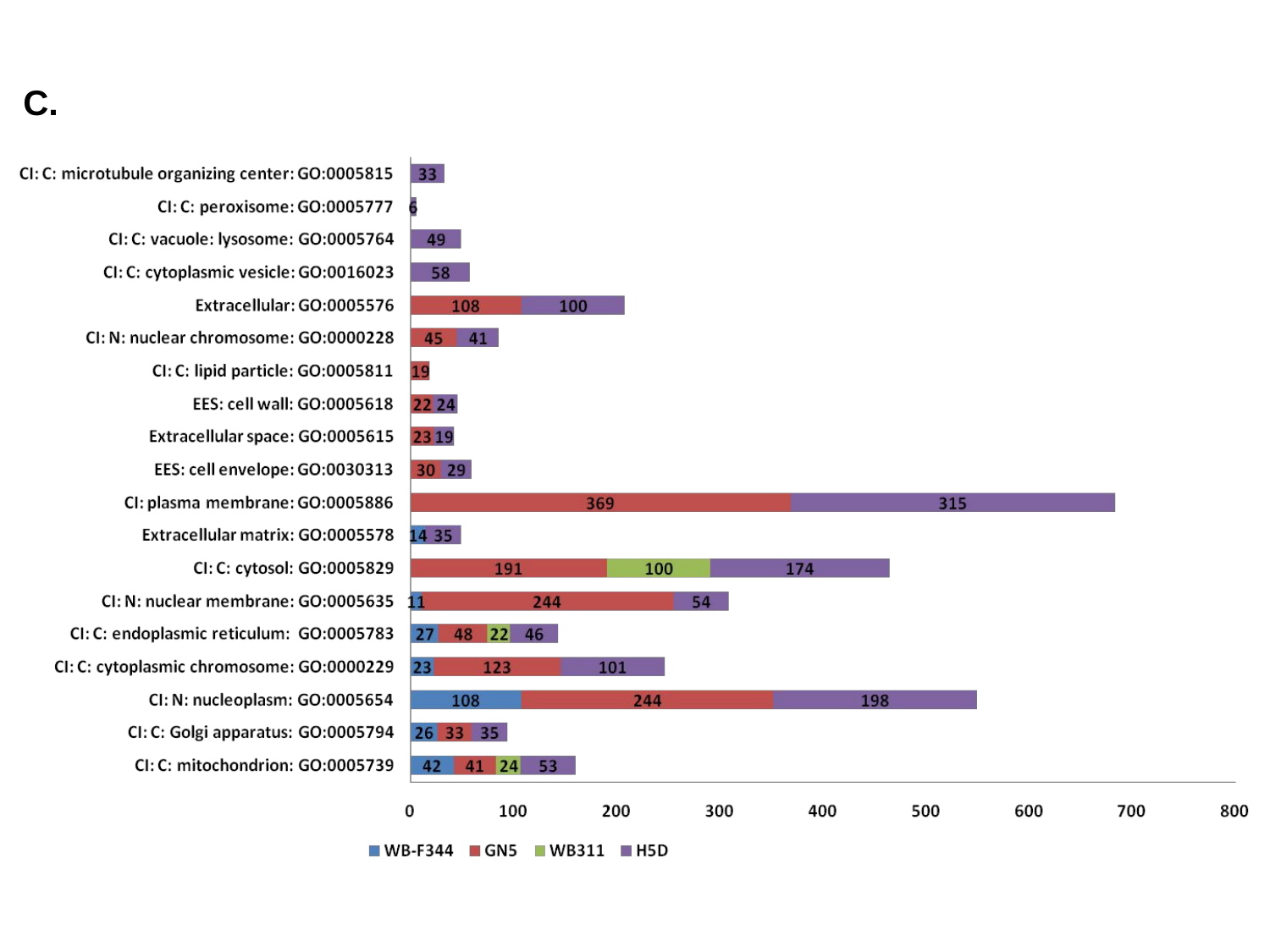

C.

Supplement: Figure S1 — Gene Ontology Analysis. Panel A. Gene ontology biological process terms responsive to rapamycin (50 nM, 24 hr) in hepatic cell lines. The bars indicate the number of differentially expressed genes per cell line for each significant Gene Ontology (GO) term sub-category. C, cell growth and/or maintenance; M, metabolism; CM, cell communication; D, development; NNNNAM, nucleobase, nucleoside, nucleotide and nucleic acid metabolism; PM, protein metabolism; T, transport. Panel B. Gene ontology molecular function terms responsive to rapamycin (50 nM, 24 hr) in hepatic cell lines. The bars indicate the number of differentially expressed genes per cell line for each significant Gene Ontology (GO) term sub-category. NAB, nucleic acid binding; CA, catalytic activity; TA, transporter activity; TFA, transcription factor activity; B, binding. Panel C. Gene ontology cell component terms responsive to rapamycin (50 nM, 24 hr) in hepatic cell lines. The bars indicate the number of differentially expressed genes per cell line for each significant Gene Ontology (GO) term sub-category. CI, cell:intracellular; C, cytoplasm; EES, external encapsulating structure. (0.42 MB PPT) [file pone.0007373.s001.ppt]

## Slide 1
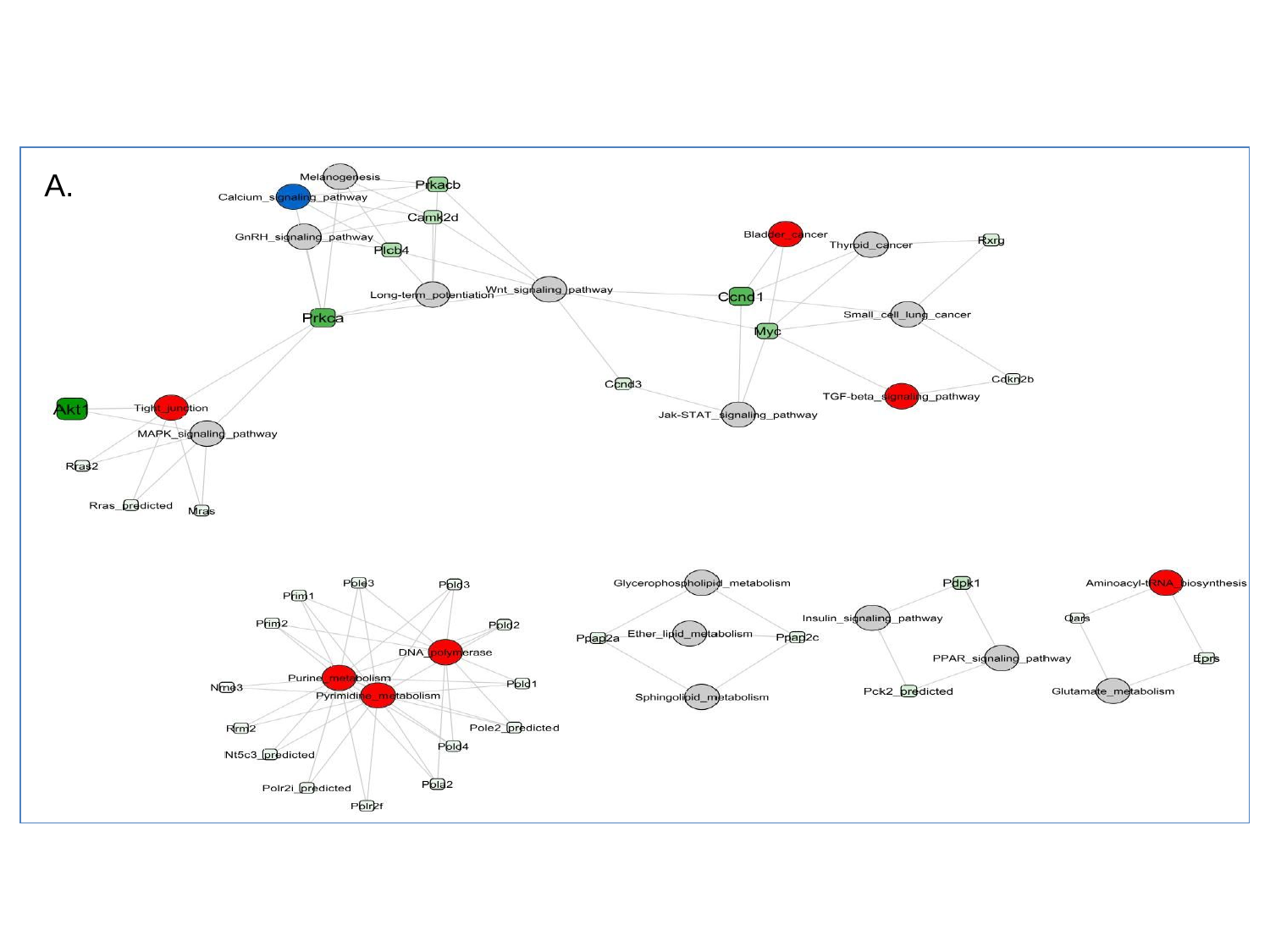

A.

## Slide 2
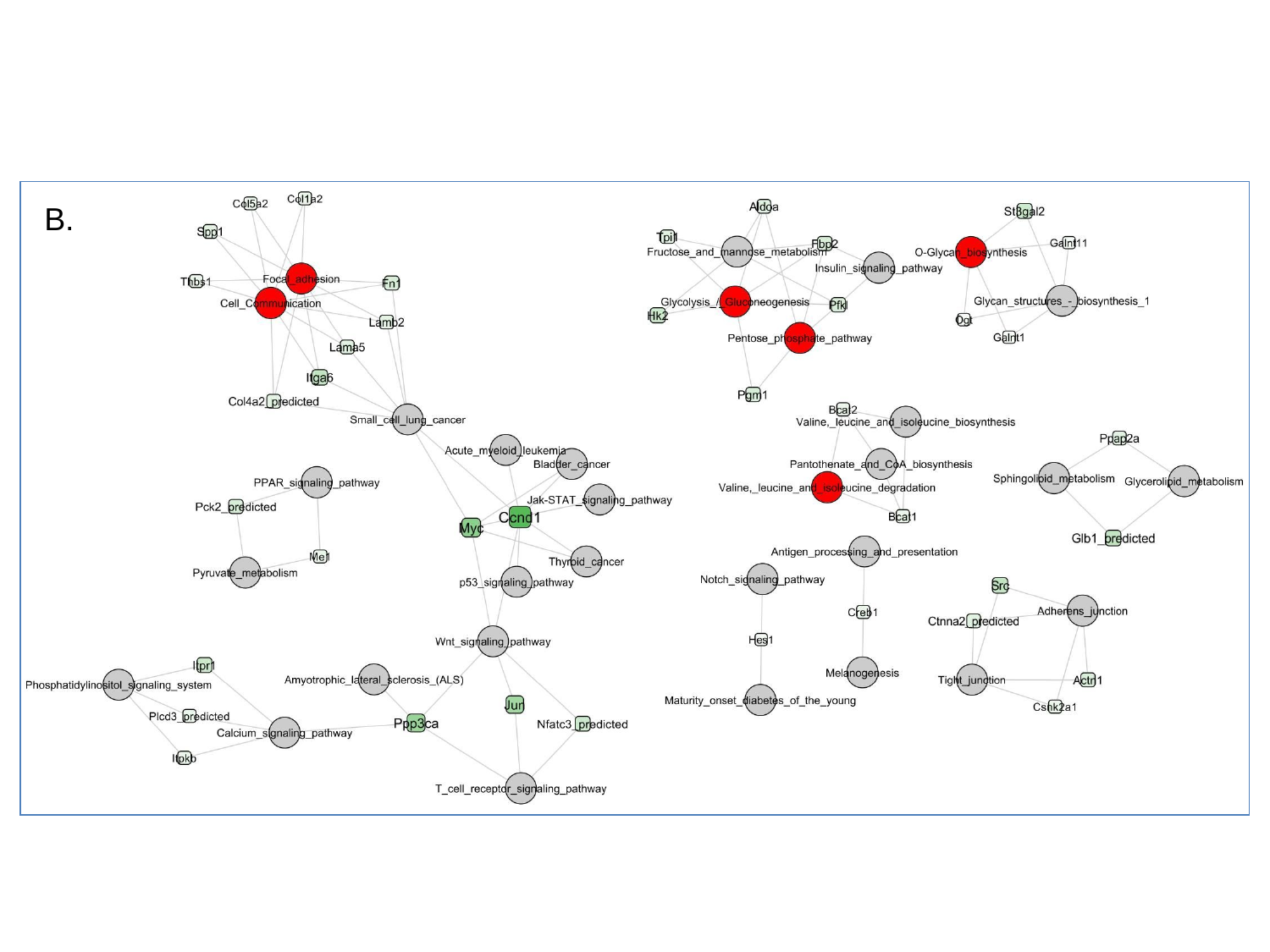

B.

## Slide 3
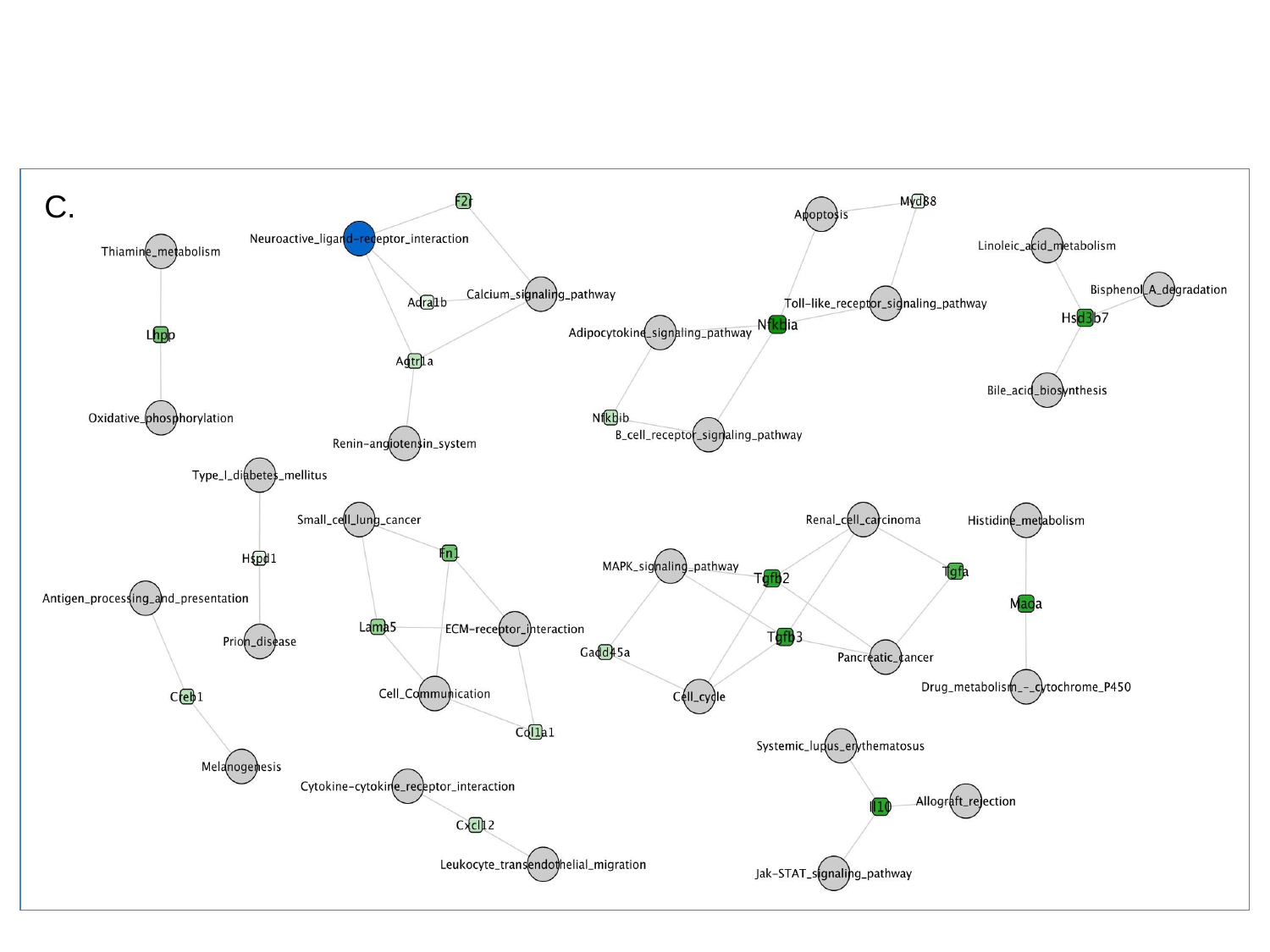

C.

## Slide 4
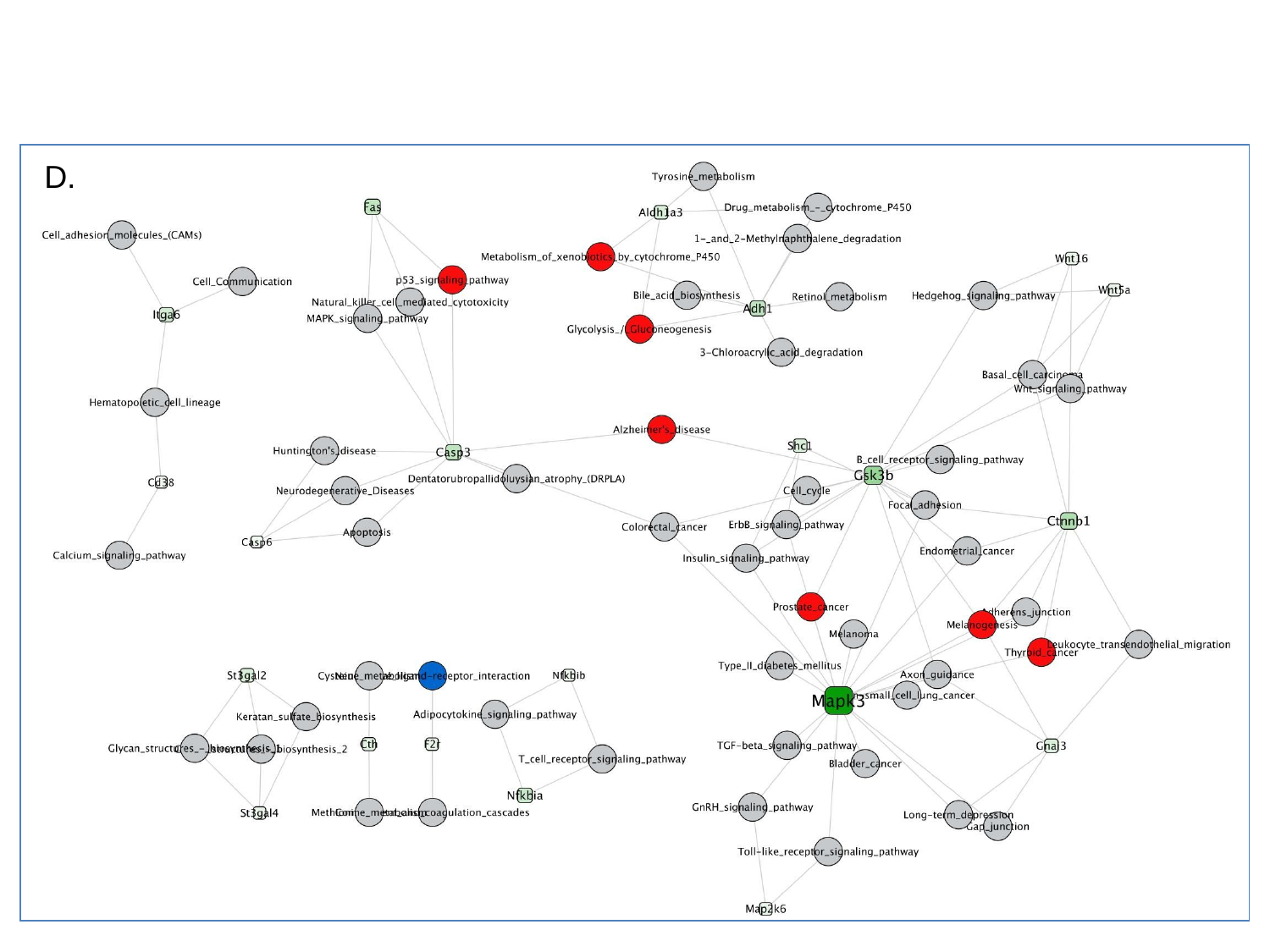

D.

Supplement: Figure S2 — Bipartite Network of Pathways Analysis. Panel A. Bipartite network of pathways and genes representing targets of mTOR in WB-F344 cells. Pathways are represented by circles and genes by squares. Gray circles indicate pathways that were not significant while red and blue circles indicate significant pathways that are overrepresented and underrepresented, respectively. The intensity of the squares with green shading indicates the degree of pathway membership ranging from light green (genes connected to few pathways) to dark green (the hubs). Pathway sub-networks are related to bladder cancer, TGF-beta signaling, tight junction, metabolism and aminoacyl-tRNA biosynthesis biological functions. The Akt, Prkca, Ccnd1 and myc genes were connected to significant pathways. Panel B. Bipartite network of pathways and genes representing targets of mTOR in WB311 cells. Symbols are those used for Figure 1A. Pathway sub-networks are related to metabolism and cellular biological functions. The Ccnd1 and myc genes were connected to pathways that were not significant. Panel C. Bipartite network of pathways and genes representing targets of mTOR in GN5 cells. Symbols are those used for Figure 1A. Overrepresented pathway sub-networks were not identified, while the neuroactive ligand receptor interaction pathway was underrepresented. The Nfkbia, Hsd3b7, Tgfb2, Tgfb3, Tgfa, and Maoa genes were connected to pathways that were not significant. Panel D. Bipartite network of pathways and genes representing targets of mTOR in H5D cells. Symbols are those used for Figure 1A. The neuroactive ligand receptor interaction pathway was underrepresented. Overrepresented pathway sub-networks are related to metabolism, Alzheimer's disease, melanogenesis, thyroid cancer and prostate cancer. The Mapk3 gene was connected to pathways that were not significant. (0.87 MB PPT) [file pone.0007373.s002.ppt]
