# Supplementary material for: Rapamycin Response in Tumorigenic and Non-Tumorigenic Hepatic Cell Lines
Source: PLoS One. 2009 Oct 9;4(10):e7373. doi: 10.1371/journal.pone.0007373 (PMC2756589; doi:10.1371/journal.pone.0007373)
Supplement: Table S2 — Gene ontology terms and associated genes sensitive to rapamycin among all four hepatic cell lines tested. MF, molecular function; BP, biological process. In the Probe ID column, the top number is the Affymetrix platform ID and the bottom is the Illumina platform ID. (0.04 MB DOC) [file pone.0007373.s004.doc]

|  |  |  | **WB-F344** | **WB311** | **H5D** | **GN5** |
| --- | --- | --- | --- | --- | --- | --- |
| **GO terms** | **Probe ID** | **Gene Symbol** | **Fold Change** | **Fold Change** | **Fold Change** | **Fold Change** |
| **MF: Catalytic activity: transferase** | 1368342_at ILMN_1369934 | Ampd3 | 1.84 | -1.5 | -1.4 | -1.26 |
|  | 1367599_at ILMN_1370511 | Atp5g1 | -1.47 | -1.26 | -1.25 | -1.2 |
| **MF: apoptosis regulator activity** | 1367899_at ILMN_1372371 | F2r | 1.76 | 1.25 | 1.22 | 1.53 |
| **BP: Cell Cycle** | 1369590_a_at ILMN_1349910 | Ddit3 | -1.74 | -1.27 | -1.47 | -1.89 |
| **BP: Carbohydrate metabolism** | 1375964_at ILMN_1358252 | Psph | -1.95 | -1.59 | -1.62 | -2.42 |
|  | 1387361_s_at ILMN_1369074 | Pgk1 | -1.37 | -1.41 | -1.3 | -1.21 |
| **BP: Energy Pathways** | 1367599_at; ILMN_1370511 | Atp5g1 | -1.47 | -1.26 | -1.25 | -1.2 |
| **BP: Cell Death** | 1369590_a_at; ILMN_1349910 | Ddit3 | -1.74 | -1.27 | -1.47 | -1.89 |
|  | 1367899_at; ILMN_1372371 | F2r | 1.76 | 1.25 | 1.22 | 1.53 |
