# Supplementary material for: Rapamycin Response in Tumorigenic and Non-Tumorigenic Hepatic Cell Lines
Source: PLoS One. 2009 Oct 9;4(10):e7373. doi: 10.1371/journal.pone.0007373 (PMC2756589; doi:10.1371/journal.pone.0007373)
Supplement: Table S3 — Pathways affected by rapamycin in hepatic cell lines. Table S3A. Results are given for the WB-F344 and WB311 cell lines (Table S3A) and for the GN5 and H5D cell lines (Table S3B). Grey highlighting denotes pathways common to all cell lines studied. Table S3C. Effect of rapamycin on genes in the glycolysis/gluconeogenesis pathway. Results are given for all four hepatic cell lines studied. (0.10 MB DOC) [file pone.0007373.s005.doc]

| **Table S3A** | | | | | | | |
| --- | --- | --- | --- | --- | --- | --- | --- |
| **WB-F344** | | | | **WB311** | | | |
| **Pathways** | **pval** | **Genes** | **Significant Genes** | **Pathways** | **pval** | **Genes** | **Significant Genes** |
| Aminoacyl-tRNA biosynthesis | 0.0416617 | 17 | 5 | Arginine and proline metabolism | 0.0070416 | 24 | 6 |
| Bladder cancer | 0.011765 | 33 | 9 | Biosynthesis of steroids | 5.90E-08 | 21 | 11 |
| Calcium signaling pathway | 0.0002686 | 164 | 6 | Carbon fixation | 0.0085873 | 18 | 5 |
| Cell cycle | 1.94E-11 | 99 | 37 | Cell Communication | 0.0388117 | 89 | 12 |
| DNA polymerase | 0.0001996 | 24 | 10 | ECM-receptor interaction | 0.0019961 | 63 | 12 |
| Folate biosynthesis | 0.0128833 | 13 | 5 | Focal adhesion | 0.0090925 | 167 | 22 |
| Fructose and mannose metabolism | 0.0397696 | 28 | 7 | Galactose metabolism | 0.0010097 | 17 | 6 |
| Galactose metabolism | 0.0103612 | 17 | 6 | Glycolysis / Gluconeogenesis | 0.003736 | 43 | 9 |
| Glycolysis / Gluconeogenesis | 0.0292545 | 43 | 10 | Neuroactive ligand-receptor interaction | 6.98E-06 | 221 | 2 |
| Inositol metabolism | 0.0385248 | 3 | 2 | Nicotinate and nicotinamide metabolism | 0.0435771 | 11 | 3 |
| Neuroactive ligand-receptor interaction | 2.79E-08 | 221 | 4 | O-Glycan biosynthesis | 0.0168238 | 14 | 4 |
| p53 signaling pathway | 4.01E-05 | 56 | 18 | Oxidative phosphorylation | 0.0295469 | 82 | 1 |
| Porphyrin and chlorophyll metabolism | 0.0302197 | 21 | 6 | Pentose phosphate pathway | 0.0065967 | 17 | 5 |
| Purine metabolism | 0.0043332 | 102 | 22 | Polyunsaturated fatty acid biosynthesis | 0.0036231 | 15 | 5 |
| Pyrimidine metabolism | 1.71E-05 | 53 | 18 | Terpenoid biosynthesis | 0.0302583 | 4 | 2 |
| Pyruvate metabolism | 0.0397696 | 28 | 7 | Valine, leucine and isoleucine degradation | 0.0095285 | 33 | 7 |
| Ribosome | 0.0284431 | 63 | 2 | Vitamin B6 metabolism | 0.0302583 | 4 | 2 |
| Streptomycin biosynthesis | 0.0398256 | 7 | 3 |  |  |  |  |
| TGF-beta signaling pathway | 0.0076333 | 70 | 16 |  |  |  |  |
| Tight junction | 0.0368075 | 114 | 21 |  |  |  |  |

| **Table S3B** | | | | | | | |
| --- | --- | --- | --- | --- | --- | --- | --- |
| **GN5** | | | | **H5D** | | | |
| **Pathways** | **pval** | **Genes** | **Significant Genes** | **Pathways** | **pval** | **Genes** | **Significant Genes** |
| Aminoacyl-tRNA biosynthesis | 1.90E-10 | 28 | 12 | Alanine and aspartate metabolism | 0.0008554 | 22 | 5 |
| Arginine and proline metabolism | 0.0493517 | 21 | 3 | Alzheimer's disease | 0.0015838 | 25 | 5 |
| Biosynthesis of steroids | 2.14E-05 | 16 | 6 | Aminoacyl-tRNA biosynthesis | 3.90E-05 | 28 | 7 |
| Biosynthesis of unsaturated fatty acids | 0.0240237 | 16 | 3 | Biosynthesis of steroids | 0.0175788 | 16 | 3 |
| Butanoate metabolism | 0.0380973 | 19 | 3 | Carbon fixation | 0.0175788 | 16 | 3 |
| Carbon fixation | 0.0240237 | 16 | 3 | Fatty acid elongation in mitochondria | 0.0384359 | 9 | 2 |
| Fatty acid elongation in mitochondria | 0.0476809 | 9 | 2 | Glutamate metabolism | 0.0322759 | 20 | 3 |
| Fructose and mannose metabolism | 0.0018043 | 23 | 5 | Glutathione metabolism | 8.46E-05 | 22 | 6 |
| Glutamate metabolism | 0.0435414 | 20 | 3 | Glycine, serine and threonine metabolism | 0.0254795 | 32 | 4 |
| Glycine, serine and threonine metabolism | 2.36E-07 | 32 | 10 | Glycolysis / Gluconeogenesis | 0.0010726 | 34 | 6 |
| Glycolysis / Gluconeogenesis | 0.0104721 | 34 | 5 | Glyoxylate and dicarboxylate metabolism | 0.0234897 | 7 | 2 |
| Neuroactive ligand-receptor interaction | 0.0457531 | 211 | 3 | Melanogenesis | 0.0174748 | 76 | 7 |
| Phenylalanine, tyrosine and tryptophan biosynthesis | 0.0293114 | 7 | 2 | Metabolism of xenobiotics by cytochrome P450 | 0.0444595 | 38 | 4 |
| Riboflavin metabolism | 0.0214958 | 6 | 2 | Neuroactive ligand-receptor interaction | 0.0064097 | 211 | 1 |
| TGF-beta signaling pathway | 0.0166335 | 67 | 7 | Nitrogen metabolism | 0.0120348 | 14 | 3 |
| Tryptophan metabolism | 0.0410081 | 33 | 4 | p53 signaling pathway | 0.0079584 | 50 | 6 |
| Urea cycle and metabolism of amino groups | 0.0039091 | 17 | 4 | Pantothenate and CoA biosynthesis | 0.0234897 | 7 | 2 |
| Valine, leucine and isoleucine biosynthesis | 0.0293114 | 7 | 2 | Prostate cancer | 0.0392196 | 71 | 6 |
| Wnt signaling pathway | 0.022157 | 110 | 0 | Reductive carboxylate cycle (CO2 fixation) | 0.0171754 | 6 | 2 |
|  |  |  |  | Thyroid cancer | 0.0464286 | 23 | 3 |
|  |  |  |  | Valine, leucine and isoleucine biosynthesis | 0.0234897 | 7 | 2 |

| **Table S3C.** | | | | |
| --- | --- | --- | --- | --- |
|  | **WB-344** | **WB311** | **H5D** | **GN5** |
| **Gene Symbol** | **Fold Change** | **Fold Change** | **Fold Change** | **Fold Change** |
| Ldha | -1.59 |  |  | -1.53 |
| **Pgk1** | **-1.37** | **-1.30** | **-1.21** | **-1.41** |
| Pfkl | -1.39 |  |  | -1.21 |
| Tpi1 |  | -1.35 | -1.23 |  |
